# Simple, standardized incorporation of genetic risk into non-genetic risk prediction tools for complex traits: coronary heart disease as an example

**DOI:** 10.3389/fgene.2014.00254

**Published:** 2014-08-01

**Authors:** Benjamin A. Goldstein, Joshua W. Knowles, Elias Salfati, John P. A. Ioannidis, Themistocles L. Assimes

**Affiliations:** ^1^Department of Medicine, Stanford University School of MedicineStanford, CA, USA; ^2^Department of Health Research and Policy, Stanford University School of MedicineStanford, CA, USA; ^3^Department of Statistics, Stanford University School of Humanities and SciencesStanford, CA, USA

**Keywords:** genetic risk scores, personalized medicine, coronary heart disease, electronic health records

## Abstract

**Purpose:** Genetic risk assessment is becoming an important component of clinical decision-making. Genetic Risk Scores (GRSs) allow the composite assessment of genetic risk in complex traits. A technically and clinically pertinent question is how to most easily and effectively combine a GRS with an assessment of clinical risk derived from established non-genetic risk factors as well as to clearly present this information to patient and health care providers.

**Materials and Methods:** We illustrate a means to combine a GRS with an independent assessment of clinical risk using a log-link function. We apply the method to the prediction of coronary heart disease (CHD) in the Atherosclerosis Risk in Communities (ARIC) cohort. We evaluate different constructions based on metrics of effect change, discrimination, and calibration.

**Results:** The addition of a GRS to a clinical risk score (CRS) improves both discrimination and calibration for CHD in ARIC. Results are similar regardless of whether external vs. internal coefficients are used for the CRS, risk factor single nucleotide polymorphisms (SNPs) are included in the GRS, or subjects with diabetes at baseline are excluded. We outline how to report the construction and the performance of a GRS using our method and illustrate a means to present genetic risk information to subjects and/or their health care provider.

**Conclusion:** The proposed method facilitates the standardized incorporation of a GRS in risk assessment.

## Introduction

As genotyping technologies become more common, the interpretation of genetic risk is becoming a bigger component of clinical decision-making. A particular challenge is the interpretation of such genetic information in the context of other clinical health information. Recently, the electronic MEdical Records and GEnomics (eMERGE) network outlined challenges and opportunities for integrating genetic data into an electronic health records (De Jager et al., 2009) system. One issue identified was the automated interpretation of genetic data (Gottesman et al., 2013; Kho et al., 2013; Marsolo and Spooner, 2013; Ury, 2013). The sheer size of genomic data provides many interpretative challenges, particularly in the age of whole genome sequencing with billions of variant base pairs, many of which are *de novo*.

Genetic Risk Scores (GRSs) are one tool for automating the rendition of one's genetic risk. They provide a means to aggregate the health related risk of a collection of genetic alleles into a single number, which can then be used for risk assessment. Using results from genome-wide association studies, one typically combines the observed (or meta-analyzed) log odds-ratio of the risk associated single nucleotide polymorphisms (SNPs). Such scores have been formulated for a variety of complex traits including coronary heart disease (CHD), diabetes, multiple sclerosis and schizophrenia (De Jager et al., 2009; Purcell et al., 2009; Thanassoulis et al., 2012). Overall, GRSs have been shown to modestly improve risk assessment using both traditional and more recently developed model performance metrics (Cook, 2007; Steyerberg et al., 2012).

We anticipate individuals will increasingly approach their physicians with questions regarding their genetic risk of common diseases as high density genetic profiling becomes progressively more routinely available. In this paper, we consider the emerging scenario where a hospital system decides to incorporate genetic data into their EHR for the purposes of clinical risk assessment. One obstacle hampering the effective incorporation of GRSs into clinical practice is the lack of clarity in how to most readily combine a GRS with a clinical risk assessment. Here, we describe a relatively straightforward method to combine genetic information at established susceptibility loci with a non-genetic risk prediction tool. We illustrate this approach in the context of CHD using a GRS constructed from the most promising association signals reported to date for this disease. We emphasize that the goal of this study is neither to validate the utility of a GRS in risk prediction nor to assess the best way to construct a GRS but rather to demonstrate how one might interpret a GRS and easily incorporate it into a clinical risk assessment. A GRS can be constructed in a variety of ways (Schrodi et al., 2014). One may select SNPs and define their respective high-risk allele either through the investigation of SNP effects within the cohort itself or within external studies that are typically much larger but not necessarily prospective in nature. One may also weigh the high-risk allele by its effect size observed internally or externally. In this study, we used the weighted approach deriving both the SNPs and weights from external sources. Lastly, we illustrate one way to present risk prediction analyses incorporating GRSs to patients and health care providers.

## Methods

### SNP selection and weighting

We selected SNPs from the most recent and largest multi–stage meta-analysis of GWAS for coronary artery disease conducted by the CARDIoGRAMplusC4D consortium to construct the GRS (CARDIoGRAMplusC4D Consortium et al., 2013). The study included 63,746 cases and 130,681 controls. The vast majority of the subjects included in this meta-analysis reported white/European ancestry. The meta-analysis added 15 new CHD susceptibility loci and confirmed nearly all loci that had previously reached genome-wide significance. The investigators also identified secondary signals at four established loci. Supplementary Table 9 of the CARDIoGRAMplusC4D manuscript lists all uncorrelated SNPs (*r*^2^ < 0.2) with an estimated FDR < 5% (CARDIoGRAMplusC4D Consortium et al., 2013). From this list, we selected the 50 SNPs identified by the consortium as validated SNPs because they had reached a genome-wide level of statistical significance in either the CARDIOGRAMplusC4D meta-analysis or in any previous GWAS.

We expect a subset of SNPs to be influencing the risk of CHD through traditional risk factors as the CARDIOGRAMplusC4D meta-analysis adjusted only for age and sex. Indeed, the CARDIoGRAMplusC4D investigators determined that 12 and 5 of these 50 SNPs likely influence CHD risk through effects on lipids and blood pressure based on their strong association with these traits in the Global Lipids Genetics Consortium and the International Consortium of Blood Pressure meta-analyses of GWAS, respectively (CARDIoGRAMplusC4D Consortium et al., 2013). For the purposes of this study, we classified these 17 SNPs as “risk factor SNPs.” The remaining 33 SNPs were classified as “non-risk factor SNPs.”

### Prospective cohort for testing genetic risk scores

We selected the AtherosclerosisRisk in Communities Study (ARIC) study to develop and test a GRS constructed with the 50 SNPs of interest. The ARIC Study is an ongoing prospective investigation of atherosclerosis and its clinical sequelae involving 15,792 white and black persons aged 45–64 years at recruitment (1987–1989). Detailed descriptions of the study designs, IRB consent process, sampling procedures, methods, definitions of cardiovascular outcomes, and approach to statistical analyses is published elsewhere (White et al., 1996; Volcik et al., 2006).

We selected ARIC for several reasons including the availability of individual level genome-wide data for all participants through the National Institutes of Health (National Human Genome Research Institute) controlled access database of Genotypes and Phenotypes (dbGaP), a prolonged follow up with > 1000 incident cases, and no overlap of incident cases with prevalent cases that were included in the CARDIoGRAMplusC4D consortium study (CARDIoGRAMplusC4D Consortium et al., 2013). The Affymetrix 6.0 array was used to genotype all participants of the ARIC study.

All white/Europeans without a history of CHD, myocardial infarction, or heart failure at baseline among the ARIC cohort subjects in dbGAP were eligible for study inclusion. Incident CHD was defined by the recording for the first time of either non-fatal or fatal myocardial infarction (“mi04,” “fatchd04”), CHD related revascularization procedure (“in_by04p”), or silent MI detected by ECG (“in_04s”).

The outcome of interest was incident CHD within 10 years. Those without a positive event who died or were lost to follow up prior to their 10th year anniversary of follow up were removed from analysis. All others were deemed event free at 10-years regardless of whether they developed incident CHD sometime after their 10 year anniversary of follow up.

### Clinical risk score assessment

We calculated two clinical risk scores (CRSs) to assess clinical risk at 10 years. The first was the well-known “external” Framingham Risk Score (FRS) for 10-year risk of CHD. The score is based on one's gender, age, total cholesterol, HDL cholesterol, blood pressure, and diabetes and smoking status. Ten-year risk of CHD was calculated using the published regression coefficients (Wilson et al., 1998). The second score was developed “internally” within the ARIC and tested and incorporated the same FRS risk factor variables using cross-validation (see below). Subjects with one or more missing FRS risk factors were excluded from the analysis.

### Imputation of ARIC raw genotype data to 1000 genomes

We imputed individual level genotype data from ARIC to the latest build of the 1000 genomes project (1 kGP) used a hidden Markov model to minimize the need to use proxy SNPs in the construction of the GRS (Abecasis et al., 2012; Howie et al., 2012). We first phased each chromosome using MaCH (v1.0.16) by running 20 rounds of the Markov sampler and considering 200 haplotypes (states) when updating each individual. We then used phased haplotypes in each chromosome and the latest release of the 1 kGPcosmopolitan panel (version 3 March 2012 release, 246 AFR + 181 AMR + 286 ASN + 379 EUR) to impute all SNPs in the cosmopolitan panel using the OpenMP protocol based multi-threaded version of Minimac (v4.6) with 20 rounds and 300 states for each chromosome. Genotyped SNPs used for imputation were restricted to those with the following features: MAF > 0.1%, missing data per SNP < 2%, and Hardy-Weinberg equilibrium (HWE) *p* > 10^−6^. Of the 841,820 autosomal genotyped markers, 543,653 passed the initial quality filters and were used for the imputation of over 37 million SNPs in ARIC. We used GTOOL (Genetics Software Suite, (c) 2007, The University of Oxford) to convert Minimac dosage files to best guess genotype calls.

### GRS construction

We calculated the GRS for an individual in the typical approach as a weighted sum of the number of high risk alleles [1].

(1) $$GRS=\sum_{i\in GRS}^{50}\omega_{i}\sum_{j=1}^{2}RA_{ij}$$

where the inside summation, RA_ij_, is the count of high risk alleles and the weight, w_i_, is the meta-analyzed log odds-ratio for SNP i. We used the corresponding “combined beta” (i.e., the beta across the stage 1 and 2 CARDIOGRAMplusC4D meta-analysis) to weigh the SNP when constructing the GRS. We carefully identified the high-risk allele for each SNP. We used the GTOOL genotype calls to count high-risk alleles for all SNPs in each individual after first dropping SNPs with a low imputation quality (*r*^2^ < 0.3).

There are two primary assumptions in such a construction. Since this summation is over marginal effects, each effect is assumed to be independent. The second is that the effects are linearly additive, i.e., there are no interactions. For the first assumption, care was taken to select SNPs that are not in linkage disequilibrium (i.e., correlated) with one another in white/European descent participants (*r*^2^ < 0.2). While the second assumption is likely violated, it is also reasonable to assume that marginal effects capture a majority of genetic risk for CHD (Zdravkovic et al., 2002; Speed et al., 2012). When using the GRS we standardize it to have a mean of 0 and standard deviation of 1.

### Combining clinical and genetic risk

We present a simple and easy way to combine one's CRS and GRS by using the following model [2]:

(2) $$\text{log}(P(CHD|\text{Clinical }\&\text{GeneticFactors}))\;=\alpha +\beta_{1}CRS+\beta_{2}GRS$$

This is a standard generalized linear model, where the outcome is a binary (0–1) indicator for incident CHD within 10 years and the predictor variables are the CRS and GRS, respectively. The CRS represents either a calculated risk due to non-genetic clinical factors (as in FRS) or a summation over multiple clinical risk factors (when using internal coefficients). We emphasize the use of a log link function instead of the more frequently used logistic link function (as in logistic regression). This allows the two coefficients of interest (β_1_ and β_2_) to represent log relative risks (RR), making the following transformation more straightforward. However, we note that using the logistic link one could perform a similar transformation. After exponentiating equation [2], we obtain:

(3) $$\begin{array}{l} P(CHD|\text{Clinical}\&\text{Genetic})=e^{\alpha +\beta_{1}CRS}\times e^{\beta_{2}GRS}\\ \;=P\left(CHD|Clinical\right)\times RR_{\left(GRS\right)}^{GRS} \end{array}$$

In the second line, we have combined the intercept (α) with the effect due to clinical factors. This is generally well captured by a CRS (like FRS) that incorporates the prevalence of disease in the general population. Since we are multiplying the estimated effects for the GRS and CRS, the primary assumption is that the GRS is linearly independent of the CRS. This assumption would potentially be violated if the GRS consisted of SNPs that were thought to act entirely or largely through effects on non-genetic clinical risk factors measured at baseline. However, the impact is mitigated by controlling for the CRS while estimating the RR for the GRS in equation [2].

Therefore, to calculate a probability of CHD based on clinical and genetic factors, we must:

1. Estimate the RR for a one-unit change in GRS on the probability of CHD within 10 years controlled for CRS.
2. For a given individual:
  - Calculate the probability of CHD based on clinical factors via a FRS or Internal Score
  - Calculate the GRS (based on equation 1) and standardize it using population mean and standard deviation (SD)
  - Multiply the probability from (a) by the RR from (1) raised to the value of standardized GRS from (b) (based on second line of Equation 3)

### Evaluation of performance of risk scores

We used 10-fold cross-validation to test both the CRS and GRS, dividing the cohort into a series of independent training and test sets. We created a series of updated risk scores:

1. A CRS based solely on the FRS (no genetic information considered)
2. A CRS based solely on the internal coefficients (no genetic information considered)
3. A CRS updated with a GRS constructed using all SNPs of interest that were either well genotyped or well imputed in ARIC.
4. A CRS updated with a GRS constructed using only “non–risk factor” SNPs among the SNPs in (3)
5. A CRS updated with a GRS constructed using only “risk factor” SNPs among the SNPs in (3)

The overall relative risk for a standardized one-unit change in GRS was estimated while incorporating the CRS (either FRS or internal). Within each of the 10-folds, the training (9/10) and test (1/10), we created a standardized score based on the mean and standard deviation from the training set. The models were estimated on the training split and applied to the test split. We used three forms of assessment. First, we calculated the c-statistic to assess discrimination of the various risk scores. Discrimination refers to a model's ability to separate subjects into distinct groups, in this case, those with CHD from those without. Secondly, we calculated the RR for a one standard deviation change in GRS. Finally, we calculated the calibration slope to assess each models overall calibration (Kramer and Zimmerman, 2007). The calibration of a model is the extent to which the predicted probability reflects the true underlying probability. The calibration slope is a more interpretable statistic than the more typical Hosmer-Lemeshow statistic, representing the degree of miscalibration (Crowson et al., 2014). A calibration slope of 1.0 indicates perfect calibration while values less than 1.0 suggest over-fitting and above 1.0 poorer calibration. For example a calibration slope of 2.0 indicates a two-fold increase in miscalibration. We chose not to assess our models using the Net Reclassification Index (NRI) or the clinical NRI due to recent concerns about the utility and validity of this metric combined with changing clinical guidelines for cardiovascular disease risk assessment (Paynter and Cook, 2012; Ridker and Cook, 2013; Goff et al., 2014; Kerr et al., 2014; Muntner et al., 2014).

In a sensitivity analysis, we repeated the above comparisons but restricted the cohort to those without prevalent diabetes. We also considered a risk prediction model using only a GRS adjusted for age and gender and no other clinical risk factors to provide a perspective on the overall impact of clinical risk factors compared to the genetic risk score. Finally, we assessed the potential for population stratification by performing a principal components analysis (PCA) with 741 ancestry informative markers (AIMs) using EIGENTRAT (Price et al., 2006) followed by a regression of CHD status onto all significant components, adjusted for the clinical factors.

All analyses were performed in R 3.0.1 (R Core Team, 2014).

### Risk reports

Using the generated information, we illustrate one means to provide a risk report about an individual's clinical and genetic risk of disease. Three key pieces of information are included:

1. The number of risk alleles
2. How the individual's GRS compares to the distribution of GRSs in a comparative population.
3. The change in one's overall risk after accounting for genetic risk

The number of risk alleles represents a simple count of the number of alleles that have been associated with an increased risk of CHD. The GRS comparison to the general population is based on the individual's standardized GRS. Finally the updated risk is calculated from equation (3). A fourth piece of information that can be included in the risk report is a statement of how the individual's change in overall risk after accounting for genetic risk influences clinical management. This may be based on some well-accepted guidelines whose recommendations can be easily and reliably automated.

## Results

### ARIC cohort exclusions

**Figure 1:**
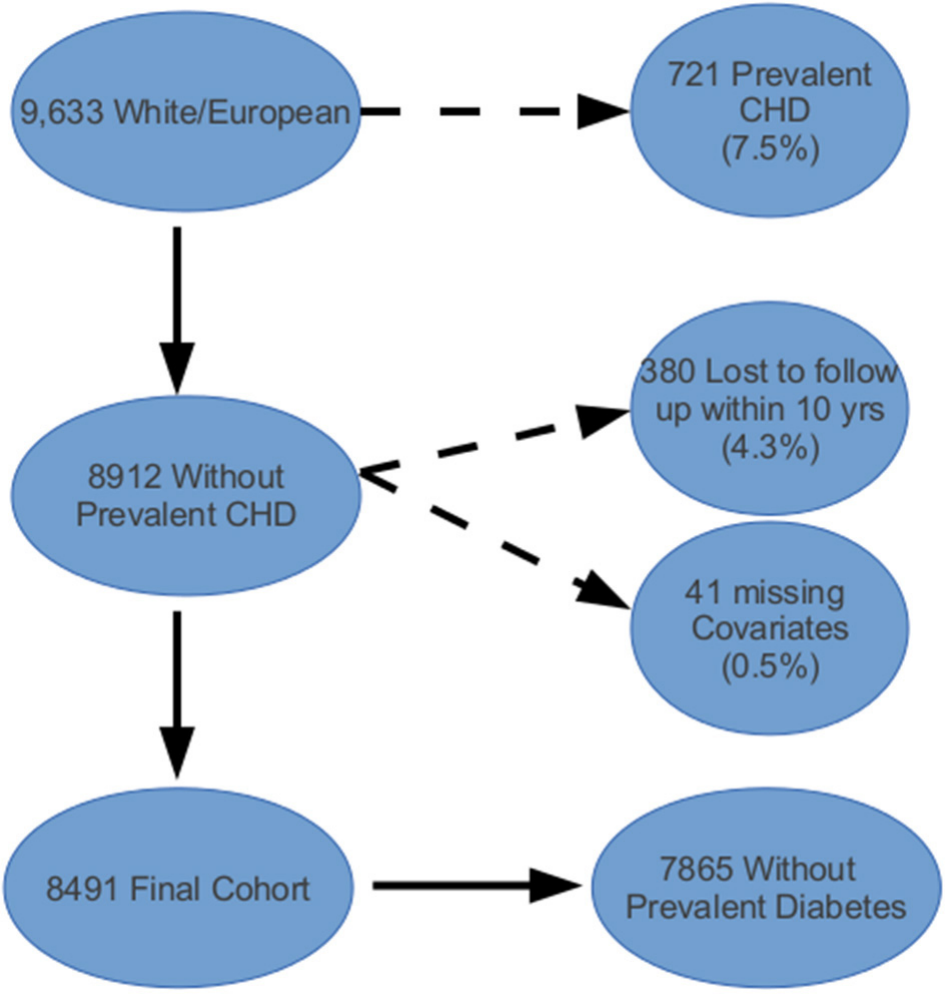
**Atherosclerosis Risk in Communities (ARIC) cohort inclusion and exclusion criteria applied to data obtained from the NCBI's database of genotypes and phenotypes (dbGAP)**.

Of the 12,771 from the ARIC cohort with phenotypic and genotypic data, 9633 (75%) were white/European (see Figure 1). Among the remaining subjects, 721 (7.5%) had a history of CHD or CHF at baseline and were excluded from further analysis. Lastly, we excluded 380 people who were lost to follow-up or died of non-CHD related factors within 10 years and 41 people with missing covariate information, comprising a final cohort of 8491. Table 1 shows the baseline characteristics for the ARIC subcohort used in our analyses. The predicted 10-year risk of developing CHD based on the FRS in this subcohort is 7.4% (interquartile range 4.3–12.3%). This predicted risk coincided very well with the observed proportion that developed CHD (7.3%).

**Table 1:** **Characteristics of the ARIC subcohort used in analyses (*n* = 8491)**.

	**mean (IQR)**
Age (years)	54 (49,59)
SBP (mm/Hg)	116 (106, 128)
DBP (mm/Hg)	71 (65, 78)
HDL (mg/dL)	48 (39, 61)
TC (mg/dL)	211 (187, 238)
	**Count (%)**
white/European	8491 (100)
Male	3848 (45)
Diabetes	626 (7.4)
**SMOKING STATUS**	
Current	2010 (24)
Former	2914 (34)
Never	3567 (42)

*IQR, inter-quartile range; SBP, Systolic Blood Pressure; DBP, Diastolic Blood Pressure; HDL, High-Density Lipoprotein Cholesterol; TC, Total Cholesterol*.

### Risk scores

The 50 SNPs of interest for construction of the GRS are listed in supplemental Table 1 along with their relationship to risk factors, weights, high risk allele based on the 1000 G reference + strand, imputation quality metrics, and genotype quality control metrics. Of the 50 SNPs, five had an estimated imputation accuracy *r*^2^ < 0.3. These five SNPs, which included two SNPs in the *APOE* locus, were dropped from the GRS. The average *r*^2^ of the remaining 45 SNPs was 0.857 (range: 0.361–0.999). The unstandardized mean value of the GRS was 3.17 (SD: 0.347) for all SNPs, 1.95 (0.307) for non-risk factor SNPs alone, and 1.22 (0.160) for risk factor SNPs alone. Interestingly, there was no difference in the unstandardized scores and standard deviations derived from the entire cohort compared to the scores derived from the subset of subjects without diabetes at baseline when considering up to three significant figures. After standardization, the mean and SD of all GRS was 0 and 1 as expected.

### Performance of risk scores and sensitivity analyses

Table 2 summarizes the c-statistics for the 8 risk scores (as well as the age and sex only scores) and the associated RR for a 1-unit change in the risk score. Adding a GRS improves overall risk discrimination. As expected, the risk score using internal weights demonstrates the best discrimination and calibration. The calibration slope statistics improved (i.e., they become smaller) with the addition of the GRS. A GRS restricted to SNPs that were not related to traditional risk factors performed essentially equally well to a GRS constructed from all SNPs combined, adding about 1 point to the c-statistic. This result suggests that the addition of CHD SNPs that are associated with CHD as well as risk factors will neither aid nor hurt risk assessment. Finally, creating a risk score only with age and sex performed worse than the risk scores with additional clinical factors. However, the improvement in both discrimination and calibration after adding the GRS is comparable to the scores with the full clinical factors.

**Table 2:** **Relative Risks and discrimination metrics for a genetic risk score derived from 50 genome wide significant susceptibility alleles for CHD in the full ARIC sample (*n* = 8491) of white/Europeans subjects**.

	**Relative Risk (95% CI)**	C-statistic^*^	**Calibration Slope**
**USING FRS FOR CLINICAL RISK SCORE**			
FRS alone	–	75.8	7.32
+ full GRS	1.29 (1.20, 1.40)	76.8	6.26
+ GRS restricted to non-risk factor SNPs	1.29 (1.20, 1.40)	76.8	6.29
+ GRS restricted to risk factor SNPs	1.06 (0.98, 1.14)	75.8	7.22
**USING INTERNAL COEFFICIENTS FOR CLINICAL RISK SCORE**			
Internal coefficients alone	–	77.3	4.34
+ full GRS	1.28 (1.19,1.38)	78.3	4.17
+ GRS restricted to non-risk factor SNPs	1.29 (1.20, 1.39)	78.3	4.18
+ GRS restricted to risk factor SNPs	1.05 (0.97, 1.13)	77.4	4.31
**USING ONLY AGE AND SEX**			
Internal coefficients alone	–	68.9	11.22
+ full GRS	1.31 (1.22,1.41)	70.4	9.26
+ GRS restricted to non-risk factor SNPs	1.29 (1.20,1.39)	70.1	9.69
+ GRS restricted to risk factor SNPs	1.11 (1.03, 1.20)	69.2	10.79

CHD, Coronary Heart Disease; ARIC, Atherosclerosis Risk in Communities; FRS, Framingham Risk score; SNPs, Single Nucleotide Polymorphism; GRS, genetic risk score;

* *performance of second model listed to first model listed*.

Table 3 summarizes the same risk score comparisons presented in Table 2 after removing 626 ARIC participants (7.4%) who reported having diabetes at baseline. We found the general trend of results to be similar to the full cohort despite a smaller sample size. There was a modest improvement in discrimination by about 1 point in the c-statistic as well as improvement in calibration.

**Table 3:** **Relative Risks and discrimination metrics for a genetic risk score derived from 50 genome wide significant susceptibility alleles for CHD in the ARIC subset of white/Europeans with no diabetes at baseline (*n* = 7865)**.

	**Relative Risk (95% CI)**	C-statistic^*^	**Calibration Slope**
**USING FRS FOR CLINICAL RISK SCORE**			
FRS alone	–	75.2	8.84
+ full GRS	1.28 (1.17, 1.39)	76.2	7.02
+ GRS restricted to non-risk factor SNPs	1.30 (1.20, 1.41)	76.3	7.22
+ GRS restricted to risk factor SNPs	1.02 (0.94, 1.11)	75.1	8.67
**USING INTERNAL COEFFICIENTS FOR CLINICAL RISK SCORE**			
Internal coefficients alone	–	76.7	6.11
+ full GRS	1.28 (1.18, 1.39)	77.6	5.39
+ GRS restricted to non-risk factor SNPs	1.30 (1.20, 1.42)	77.7	5.40
+ GRS restricted to risk factor SNPs	1.03 (0.95, 1.12)	76.6	6.00
**USING ONLY AGE AND GENDER**			
Internal coefficients alone	–	70.5	12.86
+ full GRS	1.30 (1.20,1.41)	71.8	10.49
+ GRS restricted to non-risk factor SNPs	1.28 (1.18,1.39)	71.6	10.92
+ GRS restricted to risk factor SNPs	1.10 (1.01, 1.19)	70.7	12.44

CHD, Coronary Heart Disease; ARIC, Atherosclerosis Risk in Communities; FRS, Framingham Risk score; SNPs, Single Nucleotide Polymorphism; GRS, genetic risk score;

* *performance of second model listed to first model listed*.

PCA revealed eight significant principal components. Only component 3 had a nominal association with CHD (*p* = 0.023, not corrected for number of components tested) suggesting that the addition of PCs into our model for this sample of self reported white/Europeans would not materially influence our results (Supplemental Table 2).

### Risk reports

**Figure 2:**
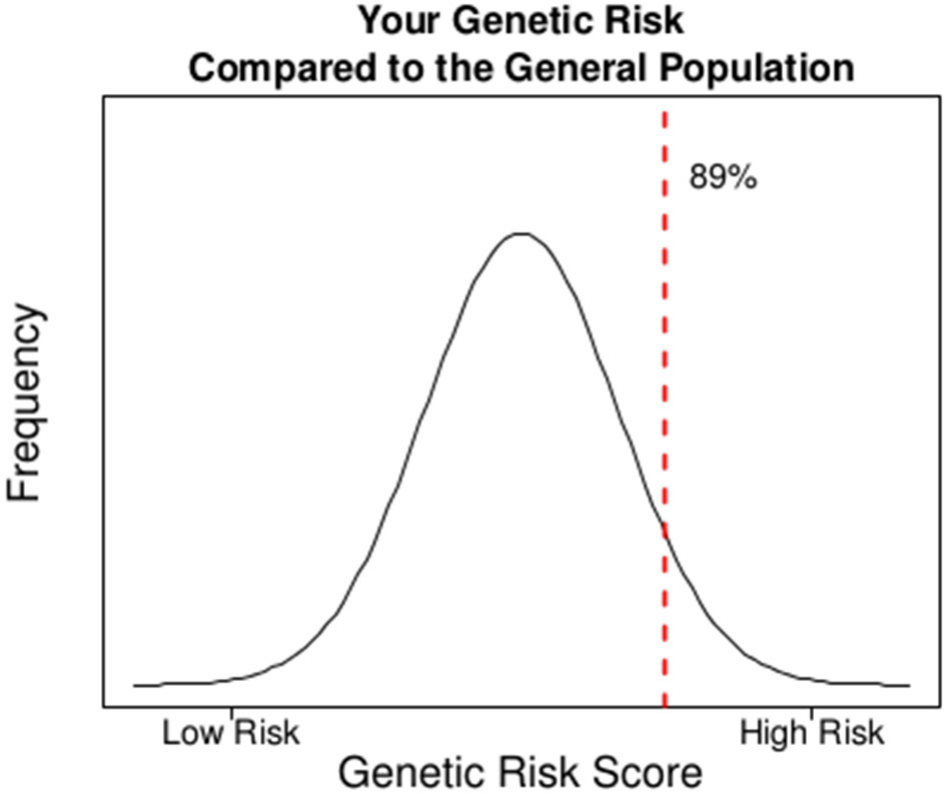
**A sample report on CHD risk for an individual in the ARIC study where the incorporation of genetic risk into the model of clinical risk potentially influences clinical management based on current guidelines**.

In Figure 2, we illustrate a sample report for an individual to show how the addition of a GRS to the model can change the risk assessment that may be used for clinical decision-making. The goal of this report would be to facilitate a conversation around the risk of CHD due to genetics above beyond the known clinical risk factors. At baseline, the participant's estimated risk of CHD at 10 years is 5.5% based on traditional Framingham risk factors. The participant carries 49 of 90 potential risk alleles resulting in a weighted standardized GRS of 1.26 which places the individual in the 89th percentile of genetic risk (i.e., only 11% of the population has a higher risk based on alleles inherited at these 45 SNPs). Combining the participant's genetic risk with their clinical risk results in a final predicted risk of CHD of 7.6% given each SD increase in one's GRS leads to a 38% increase in risk of CHD (Table 2). This magnitude of increased risk may affect the decision to treat this patient with statins (Stone et al., 2014). Ultimately, this person did develop CHD suggesting that the upward adjustment of risk was appropriate.

## Discussion

Genetic risk assessment will become an increasingly important component of overall clinical risk assessment. In this context, we ask the question: how can one most easily and effectively incorporate a GRS into an existing clinical risk assessment of a complex trait without compromising effectiveness? We present a straightforward means to combine genetic risk with clinical risk for a given disease where large-scale cohorts with prolonged follow up exist and can be used to evaluate novel biomarkers. Our approach requires knowing only three pieces of information: (1) an individual's GRS, (2) an individual's CRS, and (3) the RR associated with a 1-unit change in standardized GRS within the cohort. Recent studies demonstrate an increasing clinical utility of GRSs for CHD (Brautbar et al., 2012; Hughes et al., 2012; Thanassoulis et al., 2012, 2013; Ganna et al., 2013; Tikkanen et al., 2013). Using our method, we were able to confirm this trend and demonstrate comparable or slightly improved discrimination even when comparing our results to the subset of studies that used a GRS constructed with a similar set of SNPs (Brautbar et al., 2012; Hughes et al., 2012; Thanassoulis et al., 2012; Ganna et al., 2013; Thanassoulis et al., 2013; Tikkanen et al., 2013). We should stress that evidence in the form of a well-executed clinical trial that clearly demonstrates the value of a GRS in improving CHD outcomes does not yet exist (Ioannidis and Tzoulaki, 2010). Thus, we are not endorsing or negating the use of any specific GRS in the primary prevention of CHD on the basis of our results. Ongoing trials are examining the ability of information from GRS to improve outcomes (Knowles et al., 2012; Grant et al., 2013).

Our approach makes the simplifying assumption that the GRS is largely independent of the CRS. This assumption appears reasonable when one reliably restricts SNPs included in the GRS to those influencing risk independent of variables included in the CRS. We tested this assumption by creating two subset GRSs, one restricted to SNPs associated with risk factors and one restricted to SNPs that appear to influence risk of CHD independent of all established risk factors. The non-risk factor GRS performed noticeably better than the risk factor GRS confirming the consequence of grossly violating this assumption. However, we detected no notable difference between the non–risk factor GRS compared to the full GRS. Thus, our approach appears robust to small violations of this assumption. This confirms others' and our experiences with GRSs that they are fairly robust to alternative constructions (Purcell et al., 2009; Simonson et al., 2011).

An important consideration is the construction of the CRS. We suspect that the ability to derive and make use of such internal coefficients will be facilitated by the increasing availability of EHR with prolonged follow up of individuals receiving care as members of a large-scale health maintenance organization (Ollier et al., 2005; Palmer, 2007; Hoffmann et al., 2011a,b; Kaufman et al., 2012). As expected, the use of internal coefficients led to a slightly more effective CRS compared to the FRS that was developed in a different cohort than ARIC. Despite this observation, we observed a negligible difference in the RR suggesting that perhaps under some circumstances one can develop a GRS using an internal CRS and apply it successfully in other cohorts (or vice-versa). We also note that while the GRS improves calibration, the risk scores overall are still poorly calibrated (> 1), particularly the one using the FRS. This reflects other work that has shown that the external coefficients applied to new populations can often lead to poorly calibrated models (Ridker and Cook, 2013). Finally, the risk score using only age and sex, not surprisingly, performed the worst. Moreover, the improvement in both discrimination (68.9 vs. 77.3) and calibration (11.22 vs. 4.34) after adding additional clinical factors is much greater than after the addition of a GRS highlighting the relative importance of clinical factors collectively at this point in time over the GRS in risk assessment for CHD. However, one should not automatically assume that the current GRS is not clinically useful given its ΔAUC as it is in the same range as that seen for the addition of any single modifiable traditional risk factor to a model that includes all other traditional risk factors.

Several steps need to be followed in reporting of a GRS for a trait using our method to facilitate its testing in additional populations or to easily disseminate its use. First, the cohort in whom the GRS was derived including the age range, sex distribution, risk factor profile, and the ethnicity of its members must be clearly described. The GRS we present here is most relevant to white/Europeans in the age range of 45 to 64 and free of CHD at the time of clinical risk assessment given the eligibility criteria of the ARIC study and the fact that the SNPs used in the GRS were derived from large-scale case-control studies that included subjects in the same race/ethnic group and age range (The ARIC Investigators, 1989; CARDIoGRAMplusC4D Consortium et al., 2013). A different sets of SNPs with different weights will likely be necessary for different race/ethnic groups and possibly different age ranges although we expect substantial overlap across race/ethnic groups in the genomic regions contributing at least one SNP to the GRS (Knowles et al., 2012; Ntzani et al., 2012). Second, one must reliably identify and report which allele was coded as the high-risk allele as this allele is not necessarily the minor allele. Errors in this context due to inadvertent strand flipping either in the original study reporting the susceptibility variant or in the construction of the GRS may have a profound negative impact on the performance of the GRS. Third, the effect estimate for each SNP (generally a log odds ratio) used in the weighting of the GRS should be clearly presented. Lastly, the relative risk for a one-unit change in GRS should be calculated and clearly presented along with the mean and SD of the GRS to facilitate standardization of the score.

We suggest a means to communicate the effect on risk of someone's genetic data when combined with his or her clinical data. Our presentation includes both a contextualization relative to the general population and a statement on how one's inherited variants update one's clinical risk that is based strictly on traditional non-genetic risk factor data. In ongoing clinical investigation, we have applied a similar reporting system within a cardiology clinic (Knowles et al., 2012). Such a report can easily be automated and incorporated into an EHR. Moreover, it can also easily be updated as new susceptibility SNPs are discovered and/or weights refined. Given genome wide genotyping or sequencing is likely to become routine in the near future, more research is needed to identify the optimal way to communicate this information to subjects at risk and health care providers.

Risk scores are likely to evolve over time and practice guidelines may adopt different risk scores. For example, the FRS that we used here forms the basis of the Adult Treatment Panel III (ATPIII) guidelines (2002). Recently, ACC/AHA released new cardiovascular prevention guidelines, with new categories of risk, with a change in the relevant endpoints and in the risk calculation formulas (Goff et al., 2014; Stone et al., 2014). As of this writing, there is still large controversy about the accuracy of the new calculations and the validity of the guidelines (Cook and Ridker, 2013; Ridker and Cook, 2013; Ioannidis, 2014; Muntner et al., 2014). Regardless, our proposed methods can be used to incorporate GRS in any sets of non-genetic predictive models.

In conclusion, we present a simple but effective means to combine a CRS with a GRS and illustrate one way to present such information to an individual interested in understanding how this genetic information influences their risk assessment and thus potentially their clinical management. Furthermore, we highlight information that should be included in all reports of GRSs to facilitate the timely assessment of a new GRS by other investigators in additional populations or, alternatively, to easily incorporate it into clinical practice if its efficacy is no longer in question. We expect the importance of such research to grow over time and hope that future studies will more clearly delineate the optimal way to implement a GRS and how to most effectively disseminate a well-established GRS to patients and their health care providers.

## Funding sources

Benjamin A. Goldstein is supported by an NIH career development award K25DK097279. Joshua W. Knowles is supported by an American Heart Association, National Fellow to Faculty Award, 10FTF3360005. Themistocles L. Assimes is supported by an NIH career development award K23DK088942.

### Conflict of interest statement

The authors declare that the research was conducted in the absence of any commercial or financial relationships that could be construed as a potential conflict of interest.
