# Corrigendum: Simple, standardized incorporation of genetic risk into non-genetic risk prediction tools for complex traits: coronary heart disease as an example

**DOI:** 10.3389/fgene.2015.00231

**Published:** 2015-07-07

**Authors:** Benjamin A. Goldstein, Joshua W. Knowles, Elias Salfati, John P. A. Ioannidis, Themistocles L. Assimes

**Affiliations:** ^1^Department of Biostatistics and Bioinformatics, Center for Predictive Medicine, Duke Clinical Research Institute, Duke UniversityDurham, NC, USA; ^2^Division of Cardiovascular Medicine, Stanford UniversityStanford, CA, USA; ^3^Department of Medicine, Stanford Prevention Research Center, Stanford UniversityStanford, CA, USA

**Keywords:** risk prediction, genetic risk score (GRS), electronic health records, cardiovascular diseases, coronary disease, biomarkers

The original Figure 2 did not display the full sample risk report as described in the paper. Here we illustrate how one can convey personalized genetic risk to a patient and how the inclusion of the Genetic Risk Score changes the clinical interpretation of the individual's risk.

## Funding

BG is supported by an NIH career development award K25DK097279. JK is supported by an American Heart Association, National Fellow to Faculty Award, 10FTF3360005. TA is supported by an NIH career development award K23DK088942.

**Your Risk Score**

Based on the traditional Framingham risk score, your risk of coronary heart disease over the next 10 years is approximately 5.5%.

We tested for a total of 90 possible risks variants or alleles. Out of these 90, you carry 49 variants that are associated with higher risk. Your genetic profile puts you in the 89 percentile for risk. This means 89% of the general population have a genetic risk score more favorable than you and 11% have a genetic risk score less favorable than you.

**Figure:**
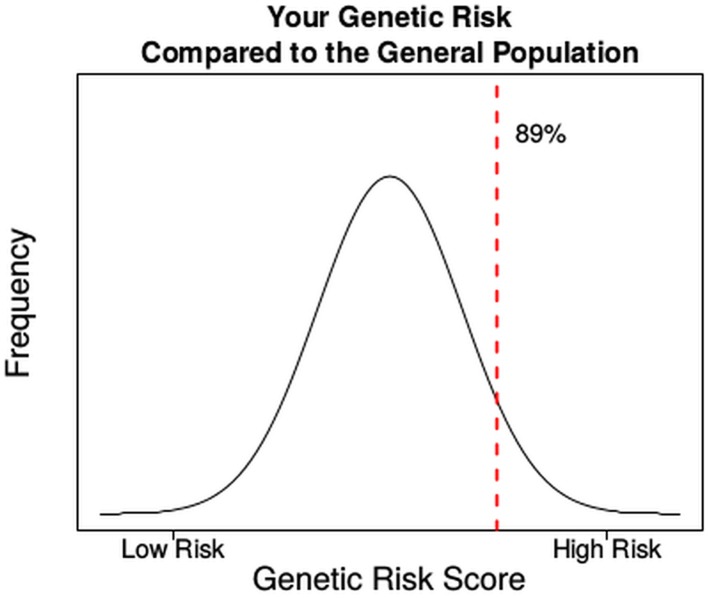

Based on the traditional Framingham risk score plus the genetic risk score, your risk of coronary heart disease over the next 10 years is approximately 7.6%.

Your 10 year risk of coronary heart disease risk is ≥7.5% when considering your genetic risk. This information may be discussed with your physician in terms of what would be recommended as most appropriate management given your estimated risk.

## Conflict of interest statement

The authors declare that the research was conducted in the absence of any commercial or financial relationships that could be construed as a potential conflict of interest.

